# Molecular detection and genotyping of pathogenic protozoan parasites in raw and treated water samples from southwest Colombia

**DOI:** 10.1186/s13071-018-3147-3

**Published:** 2018-10-26

**Authors:** Claudia Sánchez, Myriam Consuelo López, Luis Alejandro Galeano, Yvonne Qvarnstrom, Katelyn Houghton, Juan David Ramírez

**Affiliations:** 1 Grupo de Investigación en Materiales Funcionales y Catálisis (GIMFC), Departamento de Química, Facultad de Ciencias Exactas y Naturales, Universidad de Nariño, 520002 Pasto, Colombia; 2 Departamento de Salud Pública, Facultad de Medicina, Universidad Nacional de Colombia, 111321 Bogotá, Colombia; 3 Grupo de Investigaciones Microbiológicas-UR (GIMUR), Programa de Biología, Facultad de Ciencias Naturales y Matemáticas, Universidad del Rosario, 110111 Bogotá, Colombia; 4 Division of Parasitic Diseases and Malaria, Centers for Disease Control and Prevention (CDC), Atlanta, 30329 USA; 5 Oak Ridge Institute for Science and Education Research Participation Program, Oak Ridge, 37830 USA

**Keywords:** Protozoan parasites, Raw water, Treated water, PCR, Sequencing analysis

## Abstract

**Background:**

Protozoan parasites such as *Giardia duodenalis*, *Cryptosporidium* spp., *Cyclospora cayetanensis*, *Toxoplasma gondii* and *Entamoeba histolytica* represent a great challenge to the systems producing water for human consumption because their cystic forms are persistent in the environment and resist to the disinfection methods conventionally used for their control. In this study, we investigated the presence of these protozoan pathogens in both raw and treated water samples used for the production of drinking water in Nariño Department, southwest Colombia. We collected 110 water samples (10 lof each sample) and analyzed them with real-time PCR (qPCR). qPCR-positive samples were genotyped with PCR and DNA sequencing.

**Results:**

*Giardia duodenalis* was detected in 35/110 (31.8%) of the samples and *Cryptosporidium* spp. in 9/110 (8.2%) of the samples; no sample was positive for *T. gondii*, *E. histolytica* or *C. cayetanensis. Giardia duodenalis* was detected in samples of both raw water (Drinking Water Treatment Plants (DWTP): 47.83%;Drinking Water Rural Plants (DWRP): 18.42%) and water collected either after conventional physicochemical treatment (26.09%) or after disinfection by chlorine (50%), whereas *Cryptosporidium* spp. were only detected in raw waters (DWTP: 17.39%; DWRP: 13.16%). The two pathogens were detected in both types of treatment plants supplying water to urban areas and to rural zones. Analysis of *gdh* and *tpi* markers identified assemblages AI, AII and H of *G. duodenalis*, while analysis of the small subunit rRNA and *gp60* markers of *Cryptosporidium*-positive samples identified *C. parvum* (Subtype IIcA5G3c), *C. galli*, *C. molnari*, *Cryptosporidium* sp. genotype II of bats and *Cryptosporidium* sp. genotype VIII of birds.

**Conclusions:**

The results obtained demonstrate the presence of protozoan parasites in the water of the study region, and the need to improve the surveillance systems for these pathogens and identify the corresponding sources of contamination.

**Electronic supplementary material:**

The online version of this article (10.1186/s13071-018-3147-3) contains supplementary material, which is available to authorized users.

## Background

Water is an essential resource for life and thus access to safe water is currently considered a fundamental human right [1]. However, more than a billion people currently lack access to drinking water worldwide, presenting a risk for public health in affected regions [2]. In Colombia, on average, 78% of the population has access to drinking water; however, there are large differences in coverage between urban and rural areas, and it has been reported that around 1300 children die each year from diarrheal diseases caused by unsafe quality water consumption [3]. Nariño is a department located in southwest Colombia that has a risk index of water quality for human consumption (IRCA) of 50.27, which places it as a department at high risk within the country. Among the health problems related to water in Nariño, acute diarrheal disease has an incidence rate of 65.8 cases per 1000 inhabitants and a mortality rate of 18.2 cases per 1,000,000 inhabitants [4]. Additionally, in this department, an outbreak was recorded in 2017 with 2560 cases of acute diarrheal disease, in which it is presumed that water was the main vehicle of transmission of the causal agent (not yet identified) [5].

The most common and widespread risk associated with water is contamination by pathogenic microorganisms, such as viruses, bacteria and helminth and protozoan parasites. *Giardia* and *Cryptosporidium*, the causative agents of giardiasis and cryptosporidiosis, respectively, are the protozoan parasites most commonly associated with transmission by water. These pathogens affect not only humans, but a wide range of domestic and wild animals. Similarly, protozoan parasites such as *Entamoeba histolytica*, *Toxoplasma gondii* and *Cyclospora cayetanensis*, responsible for amebiasis, toxoplasmosis and cyclosporiasis, respectively, may also be transmitted by contaminated water sources and affect global health [6]. These protozoan parasites have been responsible for large numbers of outbreaks worldwide, in the period 2011–2016 at least 381 outbreaks caused by the transmission of water-borne parasitic protozoa were reported [7–9]. Protozoan parasites also represent a challenge to the production of water suitable for human consumption because their transmissible forms (cysts and oocysts) are highly stable and persistent in the environment, they can cross the physical barriers used to remove contaminants, and are resistant to several conventional disinfectants widely used in the treatment system of drinking water, such as chlorine and chloramines [10–12]. Finally, it must be emphasized that these pathogens can cause infection at rather low concentrations [13].

Current regulations in Colombia recently included the monitoring of *Giardia* spp. and *Cryptosporidium* spp. in the parameters for the microbiological control of water quality in the treatment plants responsible for the distribution of drinking water [14]. In Colombia, the presence of *Giardia* cysts and *Cryptosporidium* oocysts is regularly checked with immunofluorescence microscopy, according to the method validated by the United States Environmental Protection Agency in municipal drinking water (USEPA 1623). However, this method has several disadvantages and limitations, described by several authors as extensive experience in the microscopic differentiation of cystic forms and rigorous laboratory staff, high costs, subject to interference resulting from the presence of ions in the sample (manganese, iron and calcium) and it is not capable to identify species or genotypes, which is important for the determination of public health significance [15, 16]. As far as we know, to date there are no validated methodologies for the detection of *T. gondii*, *E. histolytica* and *C. cayetanensis* in water samples. Molecular biological techniques offer a methodological alternative in the study of protozoan parasites because their sensitivity and specificity are greater than those of traditional methods [10, 17]. For this reason, various studies have used these techniques to detect protozoan parasites such as *Giardia* [11, 18], *Cryptosporidium* [11, 18–21], *C. cayetanensis* [20, 22, 23] and *T. gondii* [20, 24, 25].

One of the great advantages of molecular biological techniques is that they allow the discrimination of microorganisms at the species and genotype levels, information that may be relevant in evaluating the sources of infection in humans and in the study of the potential risks posed by protozoan parasites [26]. For example, several assemblages of *G. duodenalis* (A-H) and about 37 species of *Cryptosporidium* have been described as associated with different hosts. Of these, particular genotypes of *C. parvum*, *C. hominis*, *C. andersoni*, *C. meleagridis*, *C. ubiquitum*, *C. cuniculus*, *C. suis* and *G. duodenalis* assemblages A and B are of special interest because they have been reported in water sources and may also present zoonotic potential [27, 28]. The identification of the different genotypes of these protozoan parasites in water sources can be useful to determine the possible sources of contamination through their association with the type of host they parasitize.

In this context, the aim of this study was to investigate the presence of protozoan parasites such as *G. duodenalis*, *Cryptosporidium* spp., *C. cayetanensis*, *E. histolytica* and *T. gondii* in samples of raw and treated water from water treatment plants that supply water for human consumption to urban areas and rural areas in the department of Nariño (southwest Colombia), using real-time PCR. We also identified the *Cryptosporidium* species and *G. duodenalis* assemblages with PCR and DNA sequencing.

## Methods

### Study area

**Fig. 1:**
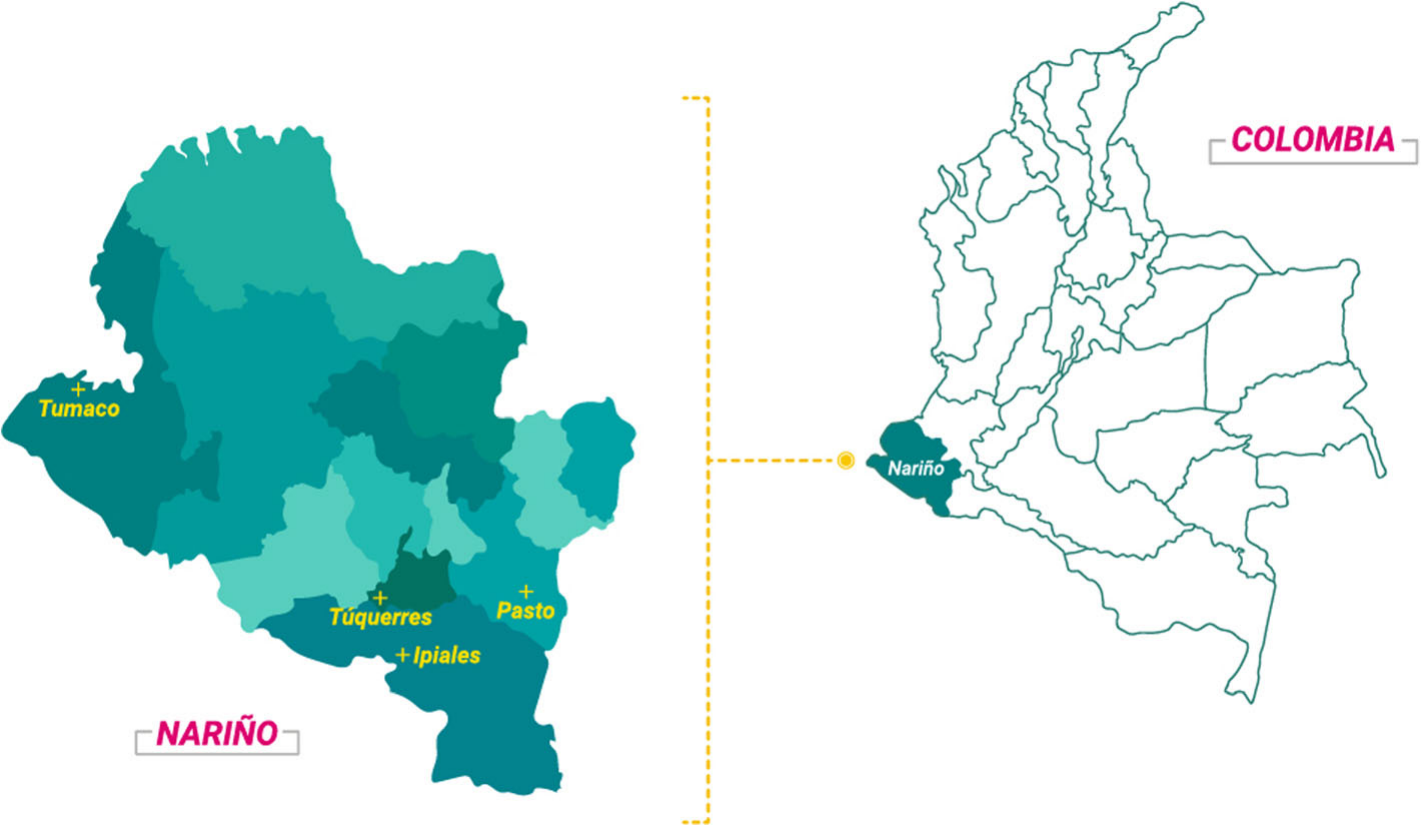
Geographical location of the sampling municipalities, all located in the Department of Nariño, southwestern Colombia

This study was performed in three Drinking Water Treatment Plants (DWTP) currently supplying drinking water to urban areas and 11 Rural Plants currently supplying water to rural areas (DWRP), in the municipalities of Pasto (1°12'52"N, 77°16'41"W; altitude: 2527 meters above sea level (masl); average temperature: 12 °C), Ipiales (0°49'44"N, 77°38'26"W; altitude: 2900 masl; average temperature: 12 °C), Túquerres (1°05'14"N, 77°37'08W; altitude: 3104 masl; average temperature: 11 °C), and Tumaco (1°48'24"N, 78°45'53W; altitude: 1 masl; average temperature: 26 °C), all located in the Department of Nariño, southwestern Colombia (Fig. 1). The three DWTPs collect the surface waters from rivers as their supply source and use a conventional physicochemical treatment to produce drinking water, including the steps of coagulation, flocculation, sedimentation, filtration and disinfection by the addition of chlorine. The DWRPs of the municipality of Ipiales use surface waters of streams as their sources of supply (DWRP-IA, DWRP-IB, DWRP-IC, DWRP-ID and DWRP-IE) and the DWRPs of the municipality of Tumaco use the surface waters of rivers (DWRP-TE) and water from underground wells (DWRP-TA, DWRP-TB, DWRP-TC, DWRP-TD and DWRP-TF) as their sources of supply. In general, DWRPs undertake minimum water treatment before consumption, consisting mainly of the addition of chlorine. However, during the sampling period, only one of the 11 DWRPs (DWRP-TF) in the study applied some type of treatment.

### Sampling

In total 117 water samples of 10 leach sample were collected (Additional file 1: Table S1). Of these, 72 water samples were collected in DWTPs at three different points: (i) 24 samples of raw water at the inlets to the plants; (ii) 24 samples after the physicochemical treatment; and (iii) 24 samples after disinfection with chlorine. The remaining 45 DWRP samples collected consisted of only raw water, because these plants did not apply any type of treatment at the times of sampling. The samples were collected on two occasions in 2016, one in March (rainy season) and the other in August-September (dry season). The sampling months were selected for convenience, based on the average monthly precipitation in the Department of Nariño, provided by the Institute of Hydrology, Meteorology, and Environmental Studies of Colombia.

### Sample processing

#### Recovery of protozoan parasites

The raw water samples were filtered through cellulose membranes with 3 μm pore size (47 mm in diameter). The water samples taken after the physicochemical treatment and after the disinfection process were also filtered through cellulose ester membranes with 1.2 μm pore size (47 mm in diameter). The membranes used in the filtration processes were washed twice with 5 ml of elution solution (0.01% Tween 80 and antifoam). To concentrate the samples, the volumes recovered from the washes were transferred to sterile polystyrene tubes and centrifuged at 1500× *g* for 15 min. Afterwards, the supernatant was discarded, leaving 1 ml of it on the pellet, which was transferred to a new sterile tube.

#### DNA extraction

The DNA was extracted from 300 μl of the previously obtained cell suspensions, using the FastDNA® SPIN Kit for Soil (MP Biomedicals, Santa Ana, USA), according to the manufacturer’s instructions. This was preceded by two cycles in a Mini-BeatBeater cell disruptor (California, USA) for 3 min and incubation on ice for 2 min to preferentially break the cystic forms of the protozoans. The DNA was eluted in 50 ml of elution buffer and stored at -20 °C until analysis. All water samples were spiked with a known concentration of a recombinant plasmid containing a sequence of an aquaporin of *Arabidopsis thaliana* as the internal control of amplification (IAC). This allowed the detection of PCR inhibitors in the samples [29].

#### Detection of parasites using real-time PCR

The DNA of *G. duodenalis*, *Cryptosporidium* spp., *T. gondii* and *E. histolytica* was detected with real-time PCR (qPCR) with endpoint detection, using primers and TaqMan probes previously reported for each of the protozoans [17, 19, 25]. The sequences of the primers and probes used are listed in Table 1. The qPCR assays were performed independently for each parasite in 96-well MicroAmp plates (Applied Biosystems, Foster City, USA), with a final reaction volume of 9 μl containing 3.5 μl of FastStart Universal Probe Master (Rox) (Roche, Basel, Switzerland), 1.0 μl of each forward and reverse primer (10 μM) and 0.2 μl of TaqMan probe (5 μM) specific for each parasite or for the internal control (IC), 0.3 μl of water, and 2.0 μl of DNA. We used the sequence of an aquaporin of *Arabidopsis thaliana* cloned into a plasmid as the internal control, as reported elsewhere [29, 30]. To detect *Cryptosporidium* spp., the final reaction volume was 10 μl because 3.0 μl of DNA was included. The samples were processed in duplicate in the Applied Biosystems 7500 system, using the default parameters and 40 cycles of amplification, except for *Cryptosporidium* spp., for which 50 cycles were used. The results of qPCR were considered negative if the cycle threshold (Ct) values were > 38. This cycle threshold was determined in a previous study by our research group. The qPCR results were considered negative if the cycle threshold values (Ct) were > 38. To corroborate the Ct value, we conducted experiments to establish the dynamic range of our assay using standards from 10,000 fg/μl to 1 fg/μl. For quantification, plasmids containing the target sequences were cloned into the pGEM-T Easy Vector System I (Promega, Madison, USA), according to the manufacturer’s instructions, and transformed into XL1-Blue Escherichia coli (Agilent Technologies, California, USA). The transformed colonies containing the plasmids were extracted by using the QIAprep Spin Miniprep Kit (Qiagen, Valencia, CA). The purified plasmid DNA was quantified by using a Nanodrop and diluted to have a concentration range of 10,000 fg/ μl to 1 fg/μl. The dynamic range established that the limit of detection was the proposed by Sanchez et al. [29]. We used DNA from *G. duodenalis*, *Cryptosporidium* spp., *T. gondii* and *E. histolytica* provided by the Parasitology Laboratory of the National University of Colombia as the positive controls in the corresponding reactions, and type I water as the negative control. We detected *C. cayetanensis* in collaboration with the Division of Parasitic Diseases and Malaria of the Centers for Disease Control and Prevention (Atlanta, USA), using a previously described method [31].

**Table 1:** Primers and probes used for the molecular detection of the protozoan parasites under study. In bold the fluorophores and quenchers

Parasite	Primer	Sequence (5'-3')	Target	Reference
*Cryptosporidium* spp.	CF	GTTTTCATTAATCAAGAACGAAAGTTAGG	*18S* rRNA	[19]
	CR	GAGTAAGGAACAACCTCCAATCTCTAG		
	CP	**6FAM**/TCAGATACCGTCGTAGTCTTAACCATAAACTATGCC/**TAMRA**		
	SSUrRNAF	AGTGACAAGAAATAACAATACAGG	*18S* rRNA	[34]
	SSUrRNAR	CCTGCTTTAAGCACTCTAATTTTC		
	GpF	GCCGTTCCACTCAGAGGAAC	*gp60*	[34]
	GpR	CCACATTACAAATGAAGTGCCGC		
*Giardia duodenalis*	GdF	CATGCATGCCCGCTCA	*18S* rRNA	[17]
	GdR	AGCGGTGTCCGGCTAGC		
	GdP	**6FAM/**AGGACAACGGTTGCAC/**MGB**		
	GDHeF	TCAACGTYAAYCGYGGYTTCCGT	*gdh*	[33]
	GDHiF	CAGTACAACTCYGCTCTCGG		
	GDHiR	GTTRTCCTTGCACATCTCC		
	AL3543	AAATIATGCCTGCTCGTCG	*tpi*	[32]
	AL3546	CAACATTITCCGCAAACC		
	AL3544	CCCCTTCATCGGIGGTAACTT		
	AL3545	GTGGCCACCACICCCGTGCC		
*Entamoeba histolytica*	EhF	GTTTGTATTAGTACAAAATGGCCAATTC	*18S* rRNA	[17]
	EhR	TCGTGGCATCCTAACTCACTTAGA		
	EhP	**6FAM/**CAATGAATTGAGAAATGACA/**MGB**		
*Toxoplasma gondii*	TgF	TCCCCTCTGCTGGCGAAAAGT	*B1* gene	[25]
	TgR	AGCGTTCGTGGTCAACTATCGATTG		
	TgP	**6FAM/**TCTGTGCAACTTTGGTGTATTCGCAG**/TAMRA**		
*Cyclospora cayetanensis*	CcF	TAGTAACCGAACGGATCGCATT	*18S* rRNA	[31]
	CcR	AAT GCC ACG GTA GGC CAA TA		
	CcP	**HEX**/CCGGCGATAGATCATTCAAGTTTCTGACC/**DABCYL**		
Internal control (Aquaglyceroporine)	CiF	ACCGTCATGGAACAGCACGTA	AQGP	[30]
	CiR	CTCCCGCAACAAACCCTATAAA		
	CiP	**VIC/**AGCATCTGTTCTTGAAGGT/**NFQ-MGB**		

#### Identification of *G. duodenalis* assemblages and *Cryptosporidium* species

Samples positive on qPCR for *G. duodenalis* and *Cryptosporidium* spp. were genotyped with conventional PCR and DNA sequencing. To identify the *G. duodenalis* assemblages, we used the following molecular markers: *gdh* (glutamate dehydrogenase) amplified with the primers GDHeF, GDHiF and GDHiR and *tpi* (triose phosphate isomerase) using the primers AL3543, AL3546, AL3544 and AL3545, as previously described [32, 33]. To identify the *Cryptosporidium* species the small subunit (SSU) rRNA marker was used, using the primers SSUrRNAF and SSUrRNAR, and subtyping of *C. parvum* was based on sequence analysis of gp60 genes as previously reported [29, 34]. The sequences of the primers are listed in Table 1. The samples were processed in a MultiGene OptiMax Thermal Cycler (Labnet, California, USA). The amplification products were verified on 2% agarose gels stained with the SYBR® Safe Stain (California, USA). The gel was visualized in an E-Gel Imager (Life Technologies, Carlsband, USA).

The PCR products were sequenced by the dideoxy-terminal method in an automated capillary sequencer (Applied Biosystems, Foster City, USA). The sequences were verified and edited in the MEGA 6.0 program [35] and then aligned with sequences from the GenBank database using the NCBI BLAST tool [36] and with reference sequences in the program ClustalW v.1.8. Phylogenetic reconstruction was performed using a maximum likelihood analysis in MEGA v6.0, using the Tamura 3-parameter method with 1000 bootstrap replicates [35]. The reference sequences considered for the *gdh* marker of *G. duodenalis* were: assemblage AI (M84604.1), assemblage AII (AY178737.1), assemblage BIII (AF069059.1), assemblage BIV (AY178739.1), assemblage C (U60982.2), assemblage D (U60986.2), assemblage E (AY178741.1), assemblage F (AB569384.1), assemblage G (AF069058.2) and assemblage H (GU176089.1). The sequence of *G. ardeae* (AF069060.2) was used as the outgroup. For the *G. duodenalis tpi* marker, the following reference sequences were used: assemblage AI (AF069556.1), assemblage AII (AF069557.1), assemblage BIII (AF069561.1), assemblage BIV (AF069560.1), assemblage C (AF069563.1), assemblage E (AY228645.1) and assemblage F (AF069558.1). The sequence of *G. microti* (AY228649.1) was used as the outgroup. For *Cryptosporidium*, the reference sequences used were: *C. andersoni* (AF093496.1), *C. baileyi* (L19068.1), *C. bovis* (AY741305.1), *C. canis* (AF112576.1), *C. fayeri* (AF159112.1), *C. felis* (AF108862.1), *C. fragile* (EU162751.1), *C. galli* (AF316624.1), *C. hominis* (AF108865.1), *C. macropodum* (AF513227.2), *C. meleagridis* (AF112574.1), *C.* cf. *molnari* (AY524773.1), *C. muris* (AB089284.1), *C. parvum* (AF112571.1), *C. ryanae* (AY587166.1), *C.* cf. *scophthalmi* (KR340588.1), *C. serpentis* (AF151376.2), *C. suis* (AF115377.1), *C. varanii* (AF112573.1) and *C. wrairi* (AF115378.1). The sequences obtained were deposited in GenBank under the accession numbers MH730625–MH730644.

### Data analysis

Descriptive statistics was used to describe the main events of interest; the results are reported as percentages and frequencies. Statistically significant associations between the detection of the parasites examined and variables, such as the place of sampling, type of water and time of sampling, were determined by logistic regression analysis, using the statistical program Statgraphics Centurion XVII (Royal Technologies, Bogota, Colombia). Differences were considered significant at *P* < 0.05.

## Results

### Detection of protozoan parasites in water samples with qPCR

A total of 117 water samples were collected, 110 of which were analyzed and seven were excluded from the study because the internal control was not amplified in any of the qPCRs. The seven samples excluded were four water samples collected in DWTPs and three water samples taken from DWRPs. Of the 110 samples analyzed, 31.82% (35/110) were positive for *G. duodenalis* and 8.18% (9/110) for *Cryptosporidium* spp. We detected both protozoan parasites *G. duodenalis* and *Cryptosporidium* together in 2.73% (3/110) of the water samples analyzed. None of the processed samples were positive for *T. gondii* (0/110), *E. histolytica* (0/110) or *C. cayetanensis* (0/110).

*Giardia duodenalis* was detected in the three DWTPs studied and in five of the 11 DWRPs (DWRP-IA, DWRP-IC, DWRP-ID, DWRP-TA and DWRP-TB). Most positive samples were found in the DWTP in Ipiales, followed by the DWTPs in Túquerres and Pasto, indicating contamination was prevalent in water from plants supplying urban areas. *Cryptosporidium* was detected in two of the three DWTPs and in four of the 11 DWRPs (DWRP-IE, DWRP-TA, DWRP-TE and DWRP-TF). We detected the greatest numbers of positive samples in the DWRPs of Tumaco, followed by the DWTP of Pasto; *Cryptosporidium* was most frequently found in treatment plants supplying rural areas. However, we found no statistically significant correlation between the sampling site and the detection of *G. duodenalis* (logistic regression, *P =* 0.1017) or *Cryptosporidium* (logistic regression, *P* = 0.4780) (Table 2).

**Table 2:** Number and percentage of positive samples for *G. duodenalis* and *Cryptosporidium* spp. for each sampling site

Site (*n*)	*G. duodenalis* (%)	*Cryptosporidium* spp. (%)
DWTP Pasto (*n* = 21)	6 (28.6)	3 (14.3)
DWTP Ipiales (*n* = 23)	13 (56.5)	1 (4.3)
DWTP Túquerres (*n* = 24)	9 (37.5)	–
DWRP Ipiales (*n* = 17)	3 (17.6)	1 (5.9)
DWRP Tumaco (*n* = 25)	4 (16.0)	4 (16.0)

*Abbreviations*: *DWTP* Drinking Water Treatment Plants*, DWRP* Drinking Water Rural Plants, *n* number of samples

**Fig. 2:**
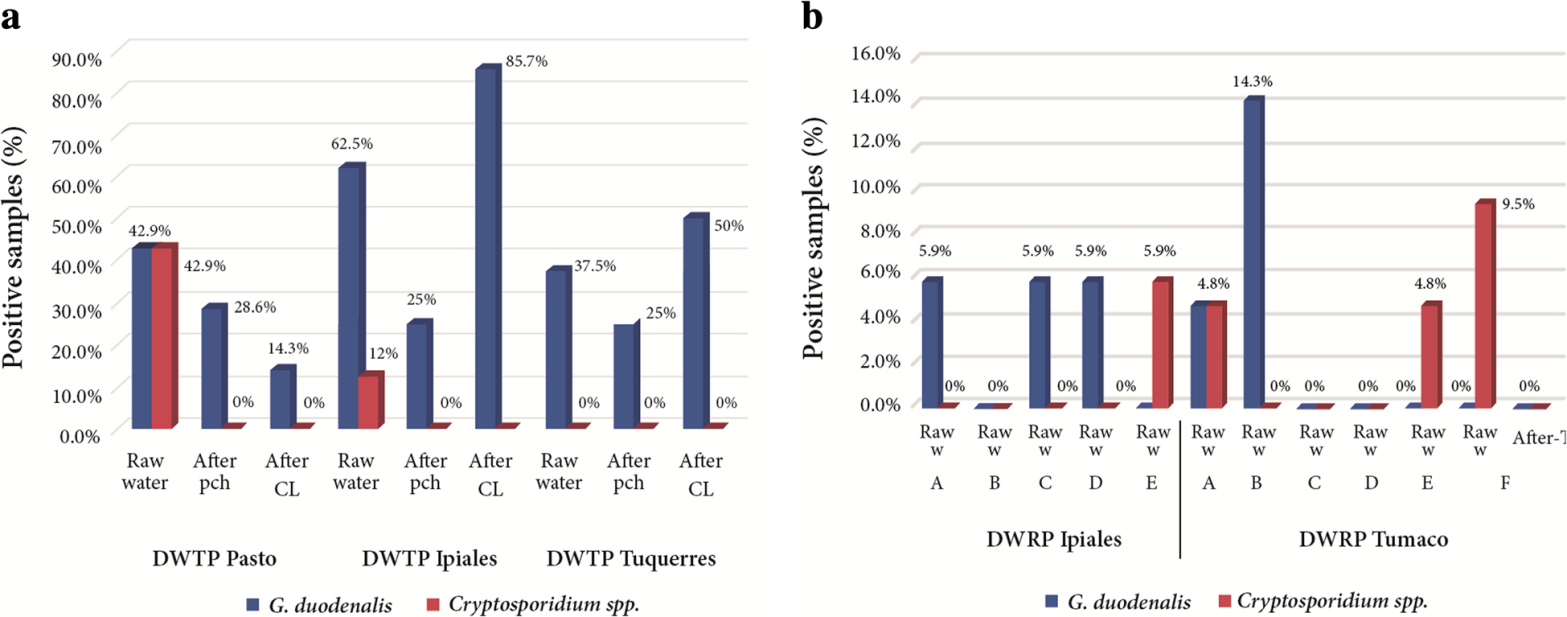
Molecular detection of *G. duodenalis* and *Cryptosporidium* spp. **a** In Drinking Water Treatment Plants currently supplying drinking water to urban areas from the municipalities of Pasto, Ipiales and Túquerres. *Abbreviations*: Raw water, water samples collected before treatment; After pch, water samples collected after the physicochemical treatment; After Cl, water samples collected after disinfection with chlorine. **b** In Rural Plants currently supplying water to rural zones from the municipalities of Ipiales and Tumaco. *Abbreviations*: Raw w, water samples collected before treatment; After T, water samples collected after treatment. DWRP Ipiales: A (Yaramal), B (La Orejuela), C (Charandu), D (Loma de Zuras) and E (Chaguaipe). DWRP Tumaco: A (Km 36), B (Cajapí), C (El Ceibito), D (Inguapí el Carmen), E (Bajo Jagua) and F (Pueblo Nuevo)

**Fig. 3:**
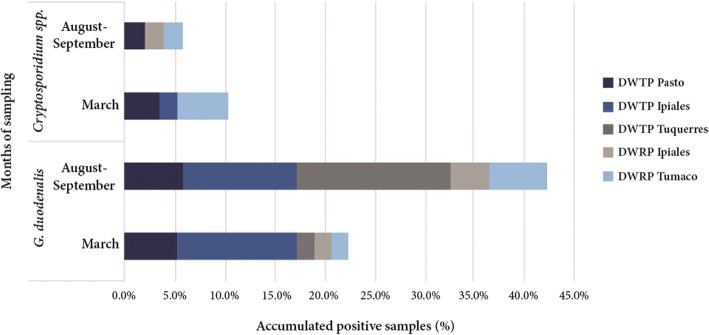
Detection of *G. duodenalis* and *Cryptosporidium* spp. in the two seasons of sampling: March (rainy season) and August-September (dry season). *Abbreviations*: DWTP, Drinking Water Treatment Plant; DWRP, Rural Plant

Of the two parasites detected, *G. duodenalis* was found in both raw and treated water samples from DWTPs (raw water: 11/23, 47.83%; after physicochemical treatment: 6/23, 26.09%; and after chlorination: 11/22, 50%) and in raw water samples from DWRPs (7/38, 18.42%). However, we detected no statistically significant relationship between the type of water and presence of this microorganism (logistic regression, *P* > 0.6835). In the DWTP of the municipality of Pasto, *G. duodenalis* was detected with greater frequency in raw water (3/7, 42.9%) than in either water collected after physicochemical treatment (2/7, 28.6%) or after chlorination (1/7, 14.3%). In the DWTPs of the municipalities of Ipiales and Túquerres, this protozoan was more frequent in the water collected after chlorination (Ipiales DWTP 6/7, 85.7%; Túquerres DWTP 4/8, 50%) than in the raw water (Ipiales DWTP 5/8, 62.5%; Túquerres DWTP 3/8, 37.5%). The fewest positive samples of *G. duodenalis* were detected in water collected after the physicochemical treatment (Ipiales DWTP 2/8, 25%; Túquerres DWTP 2/8, 25%). *Cryptosporidium* spp. were only found in raw water samples in both the DWTPs (raw water: 4/23, 17.39%) and DWRPs (5/38, 13.16%), and there was a statistically significant relationship between the type of water evaluated and this microorganism (*P* < 0.0097) (Fig. 2). *Giardia duodenalis* was detected more frequently in samples collected in the dry months of August-September (22/52, 42.31%) than in the rainy month of March (13/58, 22.41 %; *P* < 0.0078). However, the detection of *Cryptosporidium* did not vary significantly between the samples collected in August-September (3/52, 5.77%) and March (6/58, 10.34%; *P* > 0.3768) (Fig. 3).

### Identification of *G. duodenalis* assemblages and *Cryptosporidium* species

To identify the assemblages of *G. duodenalis* from the qPCR-positive samples (35/110, 31.82%), we determined the nucleotide sequences of 17 PCR products with the *gdh* marker and 28 PCR products with *tpi* marker using Sanger sequencing (the remaining samples showed only faint bands or no bands and could not be sequenced). Nucleotide sequences were determined satisfactorily for 13 samples (five with the *gdh* marker and eight with the *tpi* marker), and identified assemblages AI (1/5, 20%), AII (1/5, 20%) and H (1/5, 20%) (particularly in Tumaco) with the *gdh* marker and AI (1/8, 12.5%) and AII (4/8, 50%) with the *tpi* marker. The other samples had multiple nucleotide sequences in the same sample and therefore could not be analyzed. The same results were obtained when the samples generating these aberrant sequences were re-amplified and the PCR products sequenced again.

*Cryptosporidium* species present in the qPCR-positive water samples (9/110, 8.18%) were identified by sequencing 9 PCR products with the SSU rRNA marker. The nucleotide sequences were determined satisfactorily for seven samples, and detected *C. parvum* (1/9, 11.1%), *C. galli* (1/9, 11.1%), *C. molnari* (1/9, 11.1%), *Cryptosporidium* sp. genotype II of bats (1/9, 11.1%) and *Cryptosporidium* sp. genotype VIII of birds (3/9, 33.3%). The result of subtyping the only *C. parvum* sample showed the presence of genotype IIcA5G3c. The sequences of the two remaining samples (2/9, 22.2%) only allowed their identification to the genus level.

## Discussion

The methods used in this study allowed the recovery and detection of protozoan parasites from water samples. In the case of membrane filtration, this methodology has been used in some Latin American studies, mainly in countries such as Brazil for the recovery of protozoan parasites from various environmental samples [37]. Among its advantages are its low cost, shorter processing time in the laboratory and allowing the processing of samples with complex physicochemical composition (oils, fats, organic matter). However, a high turbidity can limit the use of this methodology due to the obstruction of the filter pores, making the use of several membranes necessary for the filtration of a single sample [15]. One limitation of our study was the use of membrane filtration. Further studies must consider the cartridge filters used in Method 1623 [1].

Regarding the qPCR, this is a technique with great potential in the detection of protozoan parasites due to its high sensitivity, a feature of great importance in environmental samples in which this type of microorganism can occur at low concentrations [11, 38]. Nevertheless, this molecular technique also has some limitations, due to the presence of PCR inhibitors that can be found in water samples and their dependence on the quality of the DNA obtained [6, 39]. In order to avoid false negatives due to the presence of inhibitors in the sample, an internal control was used in this study; this internal control identified samples that did not amplify the target DNA during the qPCR, due to the presence of substances that can act as PCR inhibitors, and therefore should be excluded from the study. In future studies it would also be important to consider the use of PCR enhancers, which could also improve the results.

With respect to the results obtained, the detection of *G. duodenalis* and *Cryptosporidium* by qPCR in water samples in this study was consistent with reports worldwide [12, 40]. *Giardia duodenalis* and *Cryptosporidium* spp. can be detected in water samples because they are widely distributed in the environment and affect both humans and a wide range of domestic and wild animals [41, 42], facilitating their transmission to water resources that are exposed to contamination [13, 18, 43]. In the DWTPs that supply water to urban areas in Nariño Department, the sources of supply are the surface waters of rivers, which are exposed to the impact of anthropogenic activities before their capture by the treatment plants. Contaminants mainly include discharges of domestic and agricultural origin, industrial effluents, runoff from waste, or the products of livestock, agriculture and human activities [44, 45].

In the DWTP of Pasto, reductions in the numbers of positive samples of *G. duodenalis* and *Cryptosporidium* were observed as the treatment of the drinking water progressed. However, this trend was not observed in the DWTPs of Ipiales and Túquerres. *Giardia duodenalis* was detected in water samples taken after the physicochemical treatment and disinfection with chlorine, at considerably higher frequencies than those in the untreated water, indicating that the procedures used in these plants must be reviewed; these findings may indicate, e.g. poor maintenance of the disinfection units, accumulation of biofilms within the pipelines or lack of optimization in these treatment facilities. One of the key factors that should be reviewed in the three DWTPs examined in this study is the physicochemical treatment (coagulation, flocculation, sedimentation and filtration), because the elimination of protozoan parasites correlates strongly with the adequate operation of each stage of this process [46]. Special attention should be paid to the steps of coagulation and flocculation [47].

In the case of *G. duodenalis*, its detection in post-treatment water samples may also be related to the resistance of the cystic forms to oxidative conventional disinfectants, such as chlorine [43, 46], which is the method applied in the plants studied. Likewise, it could be due to post-treatment contamination by cysts of this protozoan parasite during the production process of drinking water, caused by infiltrations in the treatment system through leaks, open or crossed connections, manipulation of system elements, repair of pipes, or the formation of biofilms of *Giardia* in the pipes, representing a possible source of secondary contamination of water, due to the accumulation and concentration of cysts that may occur during periods of low flow. Studies conducted in Colombia by other authors also report the presence of *Giardia* in drinking water [6, 48, 49]. However, it is important to note that in this study there was no information on the viability or infectivity of the protozoan parasites detected and therefore no conjecture could be made about the risk associated with the detection of *G. duodenalis* in the post-treatment water.

In the DWRPs of the municipalities of Ipiales and Tumaco, both *G. duodenalis* and *Cryptosporidium* spp. were detected, indicating the contamination of the natural water sources captured for human consumption in rural areas. In the DWRPs of Ipiales, the water sources used for supply could be exposed to domestic discharges, residues from crops, and the care or breeding of animals, because different anthropogenic activities are undertaken in this area. In Tumaco’s DWRPs, the groundwater wells could be contaminated because they are directly exposed to the environment and the passage of domestic and wild animals, and they are inadequately maintained. The sizes of both *Giardia* cysts and *Cryptosporidium* oocysts could also allow them to leach into the groundwater, which has previously been reported as a threat to groundwater [50, 51]. In the treatment plants that take water from rivers, the sources of contamination could be the various activities frequently performed in the rivers, including recreation, personal hygiene and laundry, and the general residual wastes of domestic origin.

Regarding the *G. duodenalis* assemblages in the qPCR-positive samples, we identified assemblage A in the DWTPs of Pasto and Ipiales. This assemblage has been reported previously in humans, livestock, companion animals, some species of marine animals and wild mammals; it is essential to consider its zoonotic potential [33, 52]. In the DWTP of Pasto, the sub-assemblage AI was identified, and in the DWTP of Ipiales, the sub-assemblages AI and AII were identified. Of these, the sub-assemblage AI has been reported predominantly in livestock and pets, and AII in humans [53]. Therefore, the sources of contamination are probably associated with these hosts. We also detected a sample containing assemblage H, so far only reported in seals and gulls [27, 52]. This represents the first description of assemblage H in Colombia or South America. Currently, the animal reservoirs of assemblage H remain undefined. Interestingly, this sample was detected in Tumaco, located at the pacific coast of the country. One plausible explanation might be that the *Giardia* assemblage H came from a seabird or marine vertebrates. However, this premise is too speculative and future studies to determine the exact frequency of this assemblage in the country and the region, as well as its hosts, are needed. Unfortunately, it was only possible to sequence a limited number of samples to identify the *G. duodenalis* assemblages, but similar results have been obtained in other studies [13, 39, 54]. The failure to amplify all qPCR-positive samples of *G. duodenalis* with nested PCR may be attributable to that the molecular marker used in the qPCR (18S rRNA) has a higher number of copies than the *gdh* and *tpi* molecular markers used in the nested PCR. However, it is also possible that some qPCR results were false positive results, due to the complexity of the environmental samples and the presence of other DNA that could cross-prime with the qPCR assays of *Cryptosporidium* spp. and *G. duodenalis* in the water samples collected and possibly with the excess of PCR cycles reported for this assay [39].

Of the *Cryptosporidium* species detected in the DWTPs, we identified *C. parvum*, *C. molnari*, *C. galli* and *Cryptosporidium* sp. genotype II of bats. *Cryptosporidium parvum* is considered one of the most widely distributed *Cryptosporidium* species, reported in more than 150 mammalian hosts, including humans, mice, cows, horses, sheep, goats, pigs and deer, and is one of the main causal agents of outbreaks of water-borne cryptosporidiosis [34, 55]. Particularly, we found the genotype IIcA5G3c, which is considered zoonotic and has been previously reported in Colombia [29]. The other species identified are considered to be host specific; *C. molnari* has mainly been described in fish, *C. galli* in birds such as chickens and finches [41], and *Cryptosporidium* sp. genotype II corresponds to the sequence of an isolate obtained from bats. It is important to remember that within the genus *Cryptosporidium*, there are several species and genotypes for which a definitive classification is still pending, so the genus is in continuous review [19, 56].

Little information is available on the relationship between the protozoan parasites detected in water resources across seasons in Latin America, where countries experience only rainy and dry seasons [16, 57]. In the present study, the number of samples positive for *G. duodenalis* was greater in August-September, which are dry months. However, there was no significant variation in the detection of *Cryptosporidium* in the dry and rainy months sampled. To better analyze this issue at a regional level, we recommend that future studies monitor these types of microorganisms over longer periods, taking into account the effects of phenomena such as El Niño and La Niña, which can alter the climatic patterns considered typical or normal for a specific region.

## Conclusions

We used molecular methodologies to determine the presence of protozoan parasites, such as *G. duodenalis* and *Cryptosporidium*, in water samples collected from the Department of Nariño. Our results provide important insights into the transmission dynamics of these protozoans in water resources. They emphasize the need for continued research and monitoring for the presence of these types of microorganisms in the water sources for human consumption, and the measures and precautions that must be considered to mitigate water contamination before its arrival at treatment plants.

## Additional file

Additional file 1: **Table S1**. Metadata information of the water samples collected and submitted to molecular detection of *Giardia duodenalis*, *Cryptosporidium*, *Entamoeba histolytica*, *Toxoplasma gondii* and *Cyclospora cayetanensis. (DOCX 24 kb)*

## Abbreviations

DWRP: Rural plants currently supplying water to rural areas
DWTP: Drinking Water Treatment Plants currently supplying drinking water to urban areas
qPCR: Real-time PCR
SSU rRNA: Small subunit ribosomal ribonucleic acid
USEPA: United States Environmental Protection Agency
gdh: Glutamate dehydrogenase
tpi: Triose phosphate isomerase
masl: Meters above sea level
