# Supplementary material for: Molecular detection and genotyping of pathogenic protozoan parasites in raw and treated water samples from southwest Colombia
Source: Parasit Vectors. 2018 Oct 26;11:563. doi: 10.1186/s13071-018-3147-3 (PMC6203992; doi:10.1186/s13071-018-3147-3)
Supplement: Supplementary file 1 — Table S1. Metadata information of the water samples collected and submitted to molecular detection of Giardia duodenalis, Cryptosporidium, Entamoeba histolytica, Toxoplasma gondii and Cyclospora cayetanensis. (DOCX 24 kb) [file 13071_2018_3147_MOESM1_ESM.docx]

**Table S1.** Metadata information of the water samples collected and submitted to molecular detection of *Giardia duodenalis*, *Cryptosporidium*, *Entamoeba histolytica*, *Toxoplasma gondii* and *Cyclospora cayetanensis*

| **March 2016** | | | **August–September 2016** | | |
| --- | --- | --- | --- | --- | --- |
| **Sample code** | **Type of sample** | **Site of sampling** | **Sample code** | **Type of sample** | **Site of sampling** |
| DWTP-P01 | Raw water | DWTP Pasto | DWTP-P13 | Raw water | DWTP Pasto |
| DWTP-P02 | Raw water | DWTP Pasto | DWTP-P14 | After physicochemical | DWTP Pasto |
| DWTP-P03 | After physicochemical | DWTP Pasto | DWTP-P15 | After disinfection | DWTP Pasto |
| DWTP-P04 | After physicochemical | DWTP Pasto | DWTP-P16 | Raw water | DWTP Pasto |
| DWTP-P05 | After disinfection | DWTP Pasto | DWTP-P17 | After physicochemical | DWTP Pasto |
| DWTP-P06 | After disinfection | DWTP Pasto | DWTP-P18 | After disinfection | DWTP Pasto |
| DWTP-P07 | Raw water | DWTP Pasto | DWTP-P19 | Raw water | DWTP Pasto |
| DWTP-P08 | After physicochemical | DWTP Pasto | DWTP-P20 | After physicochemical | DWTP Pasto |
| DWTP-P09 | After disinfection | DWTP Pasto | DWTP-P21 | After disinfection | DWTP Pasto |
| DWTP-P10 | Raw water | DWTP Pasto | DWTP-P22 | Raw water | DWTP Pasto |
| DWTP-P11 | After physicochemical | DWTP Pasto | DWTP-P23 | After physicochemical | DWTP Pasto |
| DWTP-P12 | After disinfection | DWTP Pasto | DWTP-P24 | After disinfection | DWTP Pasto |
| DWTP-T01 | After disinfection | DWTP Túquerres | DWTP-T13 | Raw water | DWTP Túquerres |
| DWTP-T02 | After physicochemical | DWTP Túquerres | DWTP-T14 | After physicochemical | DWTP Túquerres |
| DWTP-T03 | Raw water | DWTP Túquerres | DWTP-T15 | After disinfection | DWTP Túquerres |
| DWTP-T04 | After disinfection | DWTP Túquerres | DWTP-T16 | Raw water | DWTP Túquerres |
| DWTP-T05 | After physicochemical | DWTP Túquerres | DWTP-T17 | After physicochemical | DWTP Túquerres |
| DWTP-T06 | Raw water | DWTP Túquerres | DWTP-T18 | After disinfection | DWTP Túquerres |
| DWTP-T07 | After disinfection | DWTP Túquerres | DWTP-T19 | Raw water | DWTP Túquerres |
| DWTP-T08 | After disinfection | DWTP Túquerres | DWTP-T20 | After physicochemical | DWTP Túquerres |
| DWTP-T09 | After physicochemical | DWTP Túquerres | DWTP-T21 | After disinfection | DWTP Túquerres |
| DWTP-T10 | After physicochemical | DWTP Túquerres | DWTP-T22 | Raw water | DWTP Túquerres |
| DWTP-T11 | Raw water | DWTP Túquerres | DWTP-T23 | After physicochemical | DWTP Túquerres |
| DWTP-T12 | Raw water | DWTP Túquerres | DWTP-T24 | After disinfection | DWTP Túquerres |
| DWTP-I01 | Raw water | DWTP Ipiales | DWTP-I13 | Raw water | DWTP Ipiales |
| DWTP-I02 | After physicochemical | DWTP Ipiales | DWTP-I14 | After physicochemical | DWTP Ipiales |
| DWTP-I03 | After disinfection | DWTP Ipiales | DWTP-I15 | After disinfection | DWTP Ipiales |
| DWTP-I04 | Raw water | DWTP Ipiales | DWTP-I16 | Raw water | DWTP Ipiales |
| DWTP-I05 | After physicochemical | DWTP Ipiales | DWTP-I17 | After physicochemical | DWTP Ipiales |
| DWTP-I06 | After disinfection | DWTP Ipiales | DWTP-I18 | After disinfection | DWTP Ipiales |
| DWTP-I07 | After disinfection | DWTP Ipiales | DWTP-I19 | Raw water | DWTP Ipiales |
| DWTP-I08 | Raw water | DWTP Ipiales | DWTP-I20 | After physicochemical | DWTP Ipiales |
| DWTP-I09 | After physicochemical | DWTP Ipiales | DWTP-I21 | After disinfection | DWTP Ipiales |
| DWTP-I10 | After disinfection | DWTP Ipiales | DWTP-I22 | Raw water | DWTP Ipiales |
| DWTP-I11 | Raw water | DWTP Ipiales | DWTP-I23 | After physicochemical | DWTP Ipiales |
| DWTP-I12 | After physicochemical | DWTP Ipiales | DWTP-I24 | After disinfection | DWTP Ipiales |
| DWRP-IA-1 | Raw water | DWRP Ipiales Yaramal | DWRP-IA-3 | Raw water | DWRP Ipiales Yaramal |
| DWRP-IA-2 | Raw water | DWRP Ipiales Yaramal | DWRP-IA-4 | Raw water | DWRP Ipiales Yaramal |
| DWRP-IB-1 | Raw water | DWRP Ipiales La Orejuela | DWRP-IB-3 | Raw water | DWRP Ipiales La Orejuela |
| DWRP-IB-2 | Raw water | DWRP Ipiales La Orejuela | DWRP-IB-4 | Raw water | DWRP Ipiales La Orejuela |
| DWRP-IC-1 | Raw water | DWRP Ipiales Charandu | DWRP-IC-3 | Raw water | DWRP Ipiales Charandu |
| DWRP-IC-2 | Raw water | DWRP Ipiales Charandu | DWRP-IC-4 | Raw water | DWRP Ipiales Charandu |
| DWRP-ID-1 | Raw water | DWRP Ipiales Loma de Zuras | DWRP-ID-3 | Raw water | DWRP Ipiales Loma de Zuras |
| DWRP-ID-2 | Raw water | DWRP Ipiales Loma de Zuras | DWRP-ID-4 | Raw water | DWRP Ipiales Loma de Zuras |
| DWRP-IE-1 | Raw water | DWRP Ipiales Chaguaipe | DWRP-IE-3 | Raw water | DWRP Ipiales Chaguaipe |
| DWRP-IE-2 | Raw water | DWRP Ipiales Chaguaipe | DWRP-IE-4 | Raw water | DWRP Ipiales Chaguaipe |
| DWRP-TA-1 | Raw water | DWRP Tumaco Km 36 | DWRP-TA-3 | Raw water | DWRP Tumaco Km 36 |
| DWRP-TA-2 | Raw water | DWRP Tumaco Km 36 | DWRP-TA-4 | Raw water | DWRP Tumaco Km 36 |
| DWRP-TB-1 | Raw water | DWRP Tumaco Cajapí | DWRP-TB-3 | Raw water | DWRP Tumaco Cajapí |
| DWRP-TB-2 | Raw water | DWRP Tumaco Cajapí | DWRP-TB-4 | Raw water | DWRP Tumaco Cajapí |
| DWRP-TC-1 | Raw water | DWRP Tumaco El Ceibito | DWRP-TD-3 | Raw water | DWRP Tumaco Inguapí el Carmen |
| DWRP-TC-2 | Raw water | DWRP Tumaco El Ceibito | DWRP-TD-4 | Raw water | DWRP Tumaco Inguapí el Carmen |
| DWRP-TD-1 | Raw water | DWRP Tumaco Inguapí el Carmen | DWRP-TE-2 | Raw water | DWRP Tumaco Bajo Jagua |
| DWRP-TD-2 | Raw water | DWRP Tumaco Inguapí el Carmen | DWRP-TE-3 | Raw water | DWRP Tumaco Bajo Jagua |
| DWRP-TE-1 | Raw water | DWRP Tumaco Bajo Jagua | DWRP-TF-5 | Raw water | DWRP Tumaco Pueblo Nuevo |
| DWRP-TF-1 | Raw water | DWRP Tumaco Pueblo Nuevo | DWRP-TF-6 | After treatment | DWRP Tumaco Pueblo Nuevo |
| DWRP-TF-2 | After treatment | DWRP Tumaco Pueblo Nuevo | DWRP-TF-7 | Raw water | DWRP Tumaco Pueblo Nuevo |
| DWRP-TF-3 | After treatment | DWRP Tumaco Pueblo Nuevo | DWRP-TF-8 | After treatment | DWRP Tumaco Pueblo Nuevo |
| DWRP-TF-4 | Raw water | DWRP Tumaco Pueblo Nuevo |  |  |  |

* DWTP: Drinking Water Treatment Plants

* DWRP: Rural Plants
